# Diterpenylhydroquinones from Natural *ent*-Labdanes Induce Apoptosis through Decreased Mitochondrial Membrane Potential

**DOI:** 10.3390/molecules18055348

**Published:** 2013-05-10

**Authors:** Joan Villena, Alejandro Madrid, Iván Montenegro, Enrique Werner, Mauricio Cuellar, Luis Espinoza

**Affiliations:** 1 Centro de Investigaciones Biomédicas (CIB), Escuela de Medicina, Universidad de Valparaíso, Av. Hontaneda N° 2664, Valparaíso, Chile; 2 Departamento de Química, Universidad Técnica Federico Santa María, Av. España N° 1680, Valparaíso, Chile; E-Mails: alejandro.madrid@usm.cl (A.M.); ivan.montenegro@postgrado.usm.cl (I.M.); 3 Departamento De Ciencias Básicas, Campus Fernando May Universidad del Biobío, Chillán, Avda. Andrés Bello s/n casilla 447, Chile; E-Mail: ewerner@ubiobio.cl; 4 Facultad de Farmacia, Universidad de Valparaíso, Av. Gran Bretaña N° 1093, Valparaíso, Chile; E-Mail: mauricio.cuellar@uv.cl

**Keywords:** diterpenylhydroquinones, *ent*-labdanes, cancer cells, apoptosis, caspase 3 activity, mitochondrial membrane potential, *Calceolaria inamoena*

## Abstract

In this study, we examined the cytotoxic effects of seven *ent*-labdane derivatives **1**–**7** (0–100 μM) in different human cancer cell lines. Our results showed that compounds **1**–**3** exhibited significant dose-dependent inhibition on the growth of the three different human cell lines, according to the sulphorhodamine B assay and produced morphological changes consistent with apoptosis, as confirmed by Hoestch 3342 staining analysis. They induced apoptosis in various cancer cell lines, as shown by nuclear condensation and fragmentation and caspase 3 activation. Such induction was associated with the depletion of mitochondrial membrane potential. These activities led to the cleavage of caspases and the trigger of cell death process. Overall, the compounds showed potent proapoptotic effects on the two different cancer cell lines, suggesting that the compounds deserve more extensive investigation of their potential medicinal applications.

## 1. Introduction

Cancer is the second leading cause of death worldwide after cardiovascular diseases. Despite many therapeutic advances, mortality is still unacceptably high [1]. Most of the drugs used today in the clinic were first discovered from plants and microorganisms [2], therefore natural products are of increasing interest and importance to cancer patients. Specifically, for profiling the activity of anticancer drugs, the major indicators of these agents include inhibition of proliferation and induction of apoptosis in cancer cells [2]. Thus, direct manipulation of the biochemical machinery that regulates apoptosis is an interesting and new therapeutic approach to cancer [3].

Apoptosis or programmed cell death not only plays a crucial role in tissue development and homeostasis, but is also involved in a range of pathological conditions and now recognized as an important component of multi-step carcinogenesis and therapy resistance [1,4]. This physiological phenomenon represents the terminal morphological and biochemical event [5] and occurs through the activation of a cell-intrinsic suicide program. This program is carried out by internal, as well as external signals, divided into various phases terminating with signals that initiate the process leading to cell shrinkage, plasma membrane blebbing and chromatin condensation, characterized by DNA fragmentation as well as by loss of mitochondrial membrane integrity and liberation of molecules that initiate activation of intracellular proteases [3,4,6]. Mitochondria plays an important role in the apoptotic cascade by serving as a convergent center of apoptotic signals originated from both the extrinsic and intrinsic pathways [7]. The changes induced in the mitochondria membrane potential (MMP) have been previously reported to represent a determinant in the execution of cell death [8,9]. Depletion of mitochondrial membrane potential causes the opening of the mitochondria permeability transition pore, which leads to the release of apoptogenic factors, such as cytochrome c in the apoptotic cascade, and activation of caspases [10]. Caspases are a family of intracellular proteases, that are responsible directly or indirectly for the morphological and biochemical events that characterize classical apoptosis [11].

Apoptotic pathways might be significantly altered in cancer cells with respect to normal cells, and these differences might present a therapeutic window that can be exploited for the development of useful anticancer drugs [12]. Several compounds prepared employing classical organic and combinatorial chemistry have advanced into clinical trials or are already approved. Research in the last decade has revealed a promising future for apoptosis based on cancer therapies [13,14,15].

*Calceolaria inamoena* is a shrub, common to the northern subandinean part of Chile. Traditionally, various species of the genus *Calceolaria* are used as bactericidal agents, for treating stomach aches and as sweeteners [16,17,18]. Previous investigations reported about the isolation and structural determination of the mixture of *ent*-labdanes and the preparation of derivatives from *Calceolaria inamoena* [19,20,21] and the evaluation of the *in vitro* cytotoxic activities against cultured human cancer cells [22,23].

Previously a new series of antineoplastic diterpenylquinone/hydroquinones has been prepared by using labdanic diterpenoids (myrceocommunic acid derivatives) and *p*-benzoquinone or 1,4-naphthoquinone [24]. In the literature quinone/hydroquinone cytotoxicity is usually attributed to two processes [25,26]:

(1) redox cycling of quinones, resulting in generation of reactive oxygen species which can damage biomolecules, and inhibition of the mitochondrial function;
(2) electrophilic arylation of critical cellular nucleophiles, either by quinones or quinonemethides formed after bioreductive activation of quinones [27].

**Figure 1:**
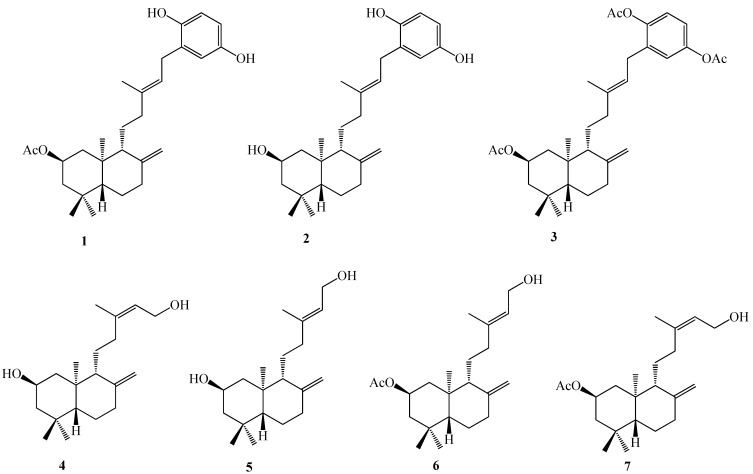
Structures of the seven compounds.

With this in mind, we decided to evaluate, for the first time, the biological activity of seven compounds previously synthesized in our laboratory (Figure 1) to potentially identify novel molecule compounds with potential anti-cancer properties. We performed this investigation using an *in vitro* bioassay based on their cytotoxic effects against cancer cells, [22,23] including DU-145 (human prostate cancer cell line), MDA-MB 231 (human breast cancer cell line) and human dermal fibroblast cell line, DHF, measured by the sulphorhodamine B dye method [23]. The active compounds were investigated to determine if they also act as apoptosis inducers as evaluated by the condensation and fragmentation of chromatin determined by Hoechst staining, caspase 3 activity and the permeabilization of mitochondrial membrane by rhodamine123 staining [15,28].

## 2. Results and Discussion

It could be inferred, based on the previous data concerning structure-activity, that in this first screening the presence of the hydroquinonic segment would initially generate oxidative stress on the tested cells causing a significant raise on cell death related the presence of allyl alcohol and free hydroxyl moieties on the terpenic decalin rings, which possess a lower activity than diterpenhydroquinones had previously shown. Also the highest apoptotic effect of the compounds, which contain in their structure the hydroquinone segment, would promote the cytotoxic ability of the terpenic decalins due, probably, to the conjugated system present in the compounds **1**–**3**, analogous to the metachromins A-H series [29] which has shown a range of cytotoxic activities against murine leukemia cells and human epidermoid carcinoma cells [30].

In previous work we reported that treatment of MDA-MB 231 and DU-145 cells with compounds **1**–**7** resulted in a marked dose-dependent cytotoxicity [23]. Among the seven compounds tested, compounds **1**–**3** displayed cytotoxic effects against one or more of the cancer cell lines. In contrast, compounds **4**–**7** presented a low cytotoxic activity against all cell lines [23], which may be related to the absence of a hydroquinone function and the inability to cross cells membrane [23]. The IC_50_ values of compounds **1**–**3** indicated that compound **2** showed the most significant cytotoxicity against the tested cancer cells [23].

**Figure 2:**
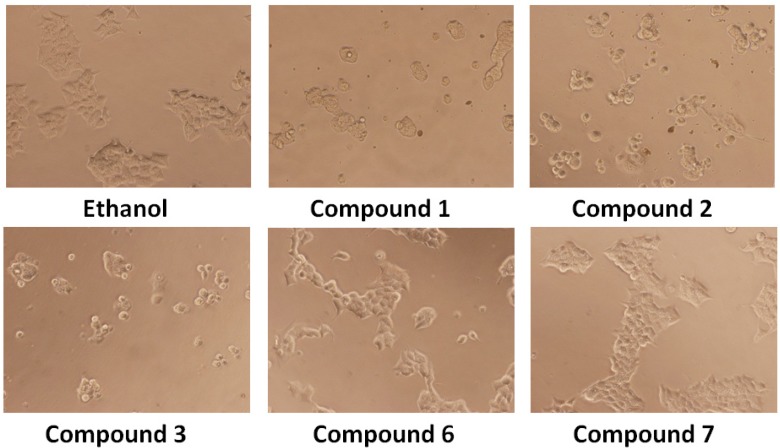
Effect of compounds on the morphology of the cells. After 24 h treatment morphological change of DU-145 cells observed under an inverted phase contrast microscope (200×).

Since compounds **1**–**3** had strong inhibitory effects on the growth of various cancer cells lines, we decided to study the effect of compounds **1**–**3** *versus* compounds **6** and **7**. First, we analyzed the appearance of morphological changes of the cells treated with 25 μM of the compounds for 24 h. Direct observation using a phase contrast microscope revealed that numerous morphological changes occurred in cells treated with compounds **1**–**3** *versus* compounds **6**–**7** as shown in Figure 2.

The cellular morphology of DU-145 was severely distorted and cells became round in shape (similar results are observed in other cell lines). Moreover, the cells showed a reduction in number, indicating an increasing progression toward cell death. The control and compound **6**–**7**-treated cells displayed normal and healthy shapes.

**Figure 3:**
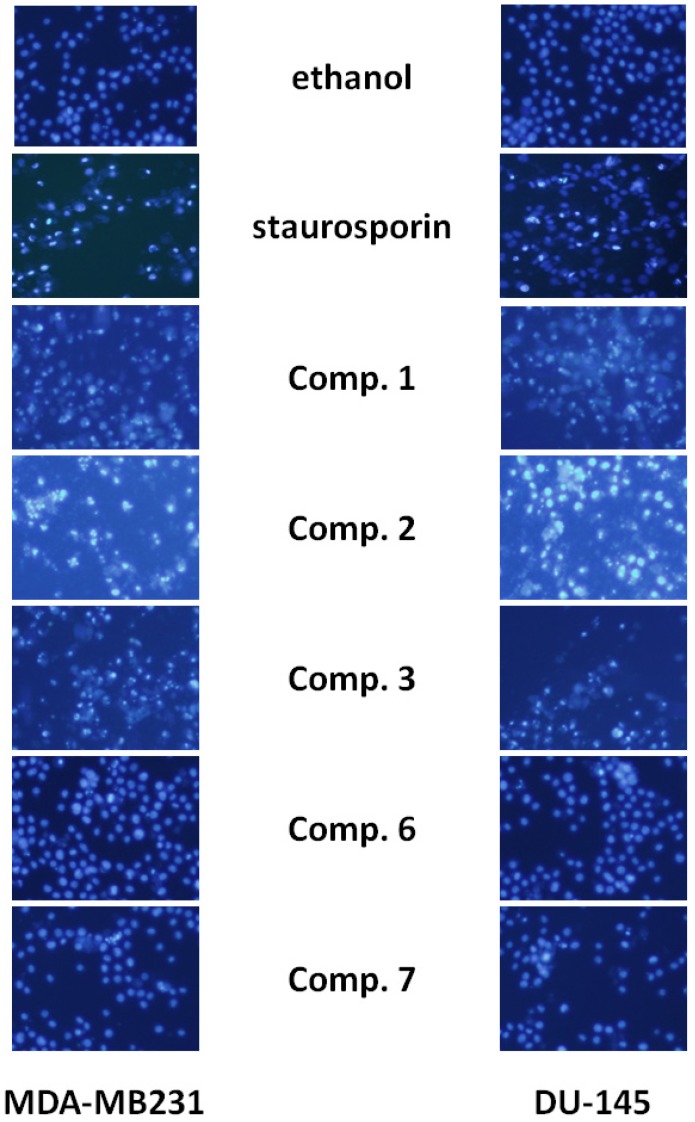
Effect of *ent*-labdanes on chromatin condensation and fragmentation. Compounds treated MDA-MB 231 and DU-145 cells were stained by Hoestch 33342 (200×). Representative photos of nuclei morphologic changes observed by fluorescent microscopy of the compounds-treated cells.

To elucidate whether compounds **1**–**3** reduced cell viability of MDA-MB 231, DU-145 and DHF cells by inducing apoptosis, cells treated with compounds **1**–**3** were examined after Hoestch 33342 staining. The nuclei changes in MDA-MB 231 and DU-145 cells were also observed under a fluorescent microscope (200×). Exposure to compounds **1**–**3** significantly affected the condensation and fragmentation nuclei in the treated cells (Figure 3).

As shown in Figure 3, control (ethanol) cells emitted a blue fluorescence with consistent intensity, indicating that the chromatin was equivalently distributed in the nuclei. Following incubation with compounds **1**–**3** for 24 h, the fluorescence light was denser and brighter compared to ethanol or compound **6**–**7-**treated cells and similar to staurosporin-treated cells. Also, the cells showed chromatin condensation and karyopyknosis, which are typical apoptotic phenomena. As shown in Table 1 treatment with compounds **1**–**3** significantly increased the number of cells with condensed and/or fragmented nuclei *versus* control-treated cells and DHF cells (* *p* < 0.01).

**Table 1:** Percentage of condensed and/or fragmented nuclei after treatment with compounds **1**–**3**, **6**, **7**, ethanol and staurosporin (* *p* < 0.01).

Compounds	MDA-MB 231	DU-145	DHF
**1**	21.3 ± 3.1 *	22.7 ± 3.5 *	10.1 ± 2.0
**2**	29.4 ± 4.3 *	21.4 ± 3.3 *	7.7 ± 1.1
**3**	18.9 ± 2.1 *	24.5 ± 3.4 *	8.4 ± 1.3
**6**	4.2 ± 0.9	6.8 ± 0.8	6.1 ± 0.9
**7**	8.7 ± 1.2	11.0 ± 1.3	8.9 ± 1.4
EtOH 1%	7.0 ± 1.3	8.6 ± 1.0	5.0 ± 1.0
Staurosporin	35.4 ± 5.1 *	42.9 ± 4.9 *	36.8 ± 5.0 *

**Figure 4:**
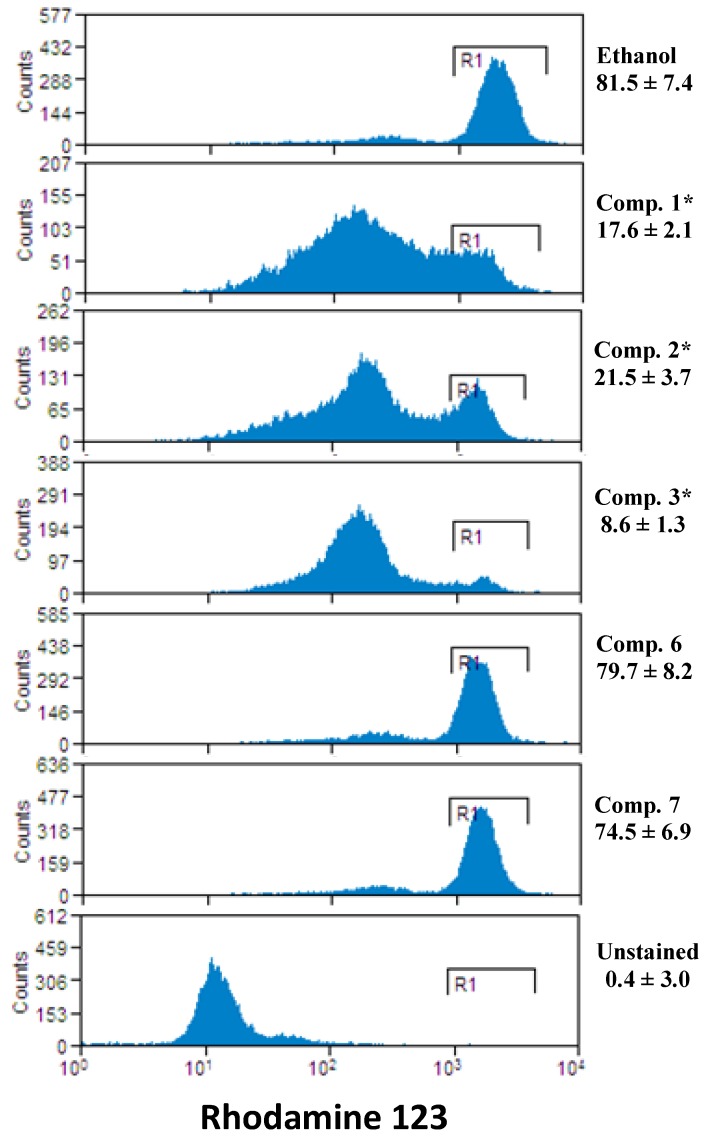
*Ent-*labdanes treatment induced changes of mitochondrial membrane permeability in MDA-MB 231. The cells were stained with rhodamine 123, and then analyzed with flow cytometry. Representative histogram is showing changes of mitochondrial membrane permeability in MDA-MB 231 cells. Also percentage of rhodamine-123 stained cells is shown. The significance was determined by Student’s t-test (* *p* < 0.001).

Mitochondria plays a crucial role in the apoptotic cascade by serving as a convergent center of apoptotic signals originated from both the extrinsic and intrinsic pathways [7]. The changes induced in the mitochondria membrane potential have been previously reported to represent a determinant in the execution of cell death [8,9]. We analyzed the possibility that the studied compounds might alter mitochondria function; the mitochondrial membrane potential was assessed. Rhodamine was employed to detect changes in mitochondrial function, since fluorescence of the dye decreases as mitochondrial membrane potential is lost [31]. As shown in Figure 4, the percentage of rhodamine 123 negative cells of the control groups was all below 15–20% of total cells. After treatment with compounds **1**–**3** (25 µM), the percentage increased significantly (* *p* < 0.001) to nearly 70–80% in cancer cells. Figure 4 showed that compounds **1**–**3** induced depletion of mitochondrial membrane permeability in cancer cells while compounds **6**–**7** have no effect on mitochondrial membrane potential. Thus, compounds **1**–**3** induced loss of mitochondrial membrane potential correlated well with cell death increase (see Figure 4).

**Figure 5:**
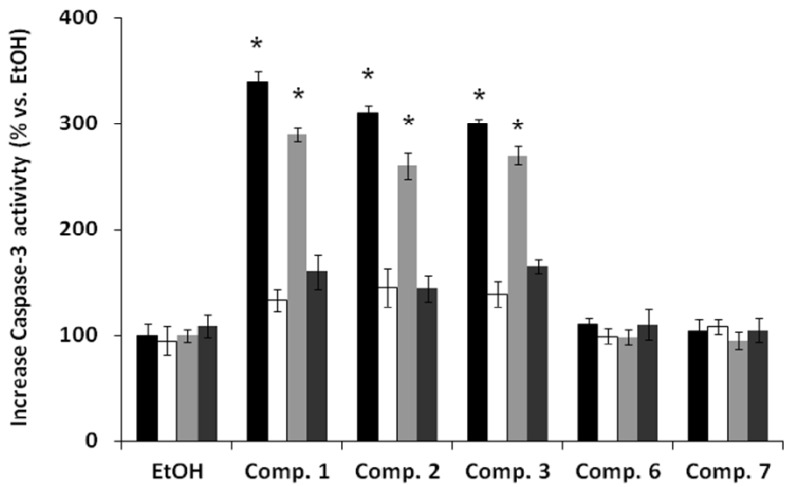
Effect of *ent*-labdanes on caspase3 activity of MDA-MB-231 (black or white columns) and DU-145 (light or dark gray columns) cells. Cells were pretreated with (white or dark gray columns) or without zVAD-fmk (black or light gray columns), a pan caspase inhibitor, and exposed to compounds **1**–**3**, **6** and **7** (25 µM) and incubated for 24 h. Values are mean ± S.D. (*n* = 3). All data is reported as the percentage change in comparison with the vehicle-treated cells, which were arbitrarily assigned 100%. * *p* < 0.01, significantly different from the vehicle-treated cells (1% ethanol in medium, that is, compound concentration = 0).

Finally, depletion of mitochondrial membrane potential causes the release of apoptogenic factors, such as cytochrome *c* in the apoptotic cascade, and activation of caspases [10]. Then we investigated the effects of compounds on caspases activity. We focused on caspase 3 which is activated by a vast number of apoptotic signals. This enzyme is a main executor of apoptosis playing a central role in its biological processing and has been reported that activation of caspase 3 is an essential event for the induction of oligonucleosomal DNA fragmentation [32]. We studied the effect of treatment with the compounds **1**–**3** with or without zVAD.fmk, a pan caspase inhibitor, on caspase 3 activities in cancer cells. As shown in Figure 5, the activity of caspase 3 in cells exposed to compounds **1**–**3** is increased versus ethanol and compounds **6**–**7** treated cells in approximately 3 times for compounds **1**–**3** in both cell lines. Moreover, when we pretreated with zVAD-fmk (white or dark gray columns) the activity of caspase 3 is decreased nearly to control cells (black or light gray columns) indicating that the effects of compounds 1–3 is specific on caspase activity.

## 3. Experimental

### 3.1. Cell Lines

The experimental cell cultures were obtained from American Type Culture Collection (Rockville, MD, USA). MDA-MB231 cell (breast cancer cell line), DU-145 (prostate cancer cell line) and dermal human fibroblast (DHF) were grown in Dulbecco’s modified Eagle’s medium (DMEM) containing 10% FCS, 100 U/mL penicillin, 100 µg/mL streptomycin and 1 mM glutamine. Cells are seeded into 96 well microtiter plates in 100 µL at plating density of 5 × 10^3^ cells/well. After 24 h incubation at 37 °C under a humidified 5% CO_2_ to allow cell attachment, the cells were treated with different concentrations of drugs and incubated for 72 h under the same conditions. Stock solution of compounds was prepared in ethanol and the final concentration of this solvent was kept constant at 1%. Control cultures received ethanol 1% only.

### 3.2. Morphological Assessment of Cell Apoptosis

#### Hoechst 33342

Morphological changes in the nuclear chromatin of cells undergoing apoptosis were revealed by a nuclear fluorescent dye, Hoescht 33342. Briefly, on 24-well chamber slides, 1 × 10^4^ cells/mL MDA-MB 231, DU-145 and DHF were cultured and exposed to 25 µM compounds for 24 h. The control group was also exposed to ethanol 1%. The cells were washed twice with phosphate buffer solution, fixed with 3.7% formaldehyde and washed again with phosphate buffer solution. Following the addition of 1 µM Hoechst 33342 (Sigma-Aldrich, Santiago, Chile), they were reacted in a dark room at room temperature for 30 min. After being washed, they were examined under an immunofluorescence microscope (Olympus IX 81 model inverted microscope).

### 3.3. Analysis of Mitochondrial Transmembrane Potential

Rhodamine 123, a cationic voltage-sensitive probe that reversibly accumulates in mitochondria was used to detect changes in transmembrane mitochondrial potential. Exponentially growing cells were incubated with *ent*-labdanes as indicated in the figure legends. Cells were labeled with 1 μM rhodamine 123 at 37 °C in cell medium for 60 min before terminating the experiment. Cells were detached from the plate after washing with ice cold PBS, the samples were analyzed by flow cytometry. Data is expressed in percentage of cells stained with rhodamine 123 [31].

### 3.4. Caspase 3 Activity Assay

Caspases activity was measured using colorimetric assay (caspase-3 kit Sigma-Aldrich, St. Louis, MO, USA) Briefly, the cells exposed to compounds were collected by centrifugation at 1000 rpm and the cells were lysed with lysis buffer (1% Triton X-100, 0.32 M sucrose, 5 mM EDTA, 10 mM Tris-HCl, pH 8.0, 2 mM dithiothreitol, 1 mM PMSF, 1 mg/mL aprotinin, 1 mg/mL leupeptin). Thereafter, the lysates were transferred to wells in a 96-well flat bottom microplate and were incubated with each substrate (final concentration of 200 µM) specific for caspase 3, at 37 °C for 1 h. Specific substrate was DEVD-pNA for caspase 3. The intensity of the developed color was read at 405 nm in a microplate reader (SpectraMax, Winooski, VT, USA). The results are expressed as a percentage of the control level.

### 3.5. Statistical Analyses

All data was expressed as percentage compared with vehicle treated control cells which were arbitrarily assigned 100%. Data was interpreted by a one-way analysis of variance followed by Dunnett’s multiple comparison tests (Sigma Stat, Jandel, San Rafael, CA, USA). For all comparisons, differences were considered statistically significant at *p* < 0.05.

## 4. Conclusions

Compounds **1**–**3** and compounds **6**, **7** derived from *Calceolaria inamoena* were tested as potential cytotoxic agents against two different cancer cell lines: prostate (DU-145 cell line) and breast (MDA-MB 231 cell line) and dermal human fibroblasts (DHF). Previous primary screens indicated IC_50_ concentration between 0 and 100 μM for compounds **1** to **3**. Among the seven, three were found to be effective against the most of the cancer cell lines. Compounds **1**–**3**, which showed cytotoxicity, were evaluated in an apoptosis assay. Compounds **1**–**3** showed a good apoptotic response against breast and prostate cancer cell culture, whereas the **6**–**7** had minor impact on induction of chromatin nuclear condensation and/or fragmentation, thereby suggesting the existence of apoptosis. Moreover the compounds **1**–**3** also depleted the mitochondrial membrane potential and increased caspase 3 activity. These compounds displayed promising activity and might be used as leads in the design and development of new anticancer drugs.
